# Application of an Integrated Statistical Design for Optimization of Culture Condition for Ammonium Removal by *Nitrosomonas europaea*

**DOI:** 10.1371/journal.pone.0060322

**Published:** 2013-04-02

**Authors:** Bao yingling, Ye zhengfang

**Affiliations:** Department of Environmental Engineering, Peking University, The Key Laboratory of Water and Sediment Sciences, Ministry of Education, Beijing, China; United States Department of Agriculture, United States of America

## Abstract

Statistical methodology was applied to the optimization of the ammonium oxidation by *Nitrosomonas europaea* for biomass concentration (*C_B_*), nitrite yield (*Y_N_*) and ammonium removal (*R_A_*). Initial screening by Plackett-Burman design was performed to select major variables out of nineteen factors, among which NH_4_Cl concentration (*C_N_*), trace element solution (*TES*), agitation speed (*AS*), and fermentation time (*T*) were found to have significant effects. Path of steepest ascent and response surface methodology was applied to optimize the levels of the selected factors. Finally, multi-objective optimization was used to obtain optimal condition by compromise of the three desirable objectives through a combination of weighted coefficient method coupled with entropy measurement methodology. These models enabled us to identify the optimum operation conditions (*C_N_* = 84.1 mM; *TES* = 0.74 ml; *AS* = 100 rpm and *T* = 78 h), under which *C_B_* = 3.386×10^8^ cells/ml; *Y_N_* = 1.98 mg/mg and *R_A_* = 97.76% were simultaneously obtained. The optimized conditions were shown to be feasible through verification tests.

## Introduction

Ammonium is the main pollutant in waste water. It can be toxic to aquatic life, cause eutrophication in receiving water, and affect chlorine disinfection efficiency [1]. Traditionally, ammonium removed from waste water is mainly based on biological technology, for it is more effective and relatively inexpensive [2]. The biological removal of ammonium is undertaken in biological nitrification/denitrificantion processes, which represents a key process in global nitrogen cycle [3]. Among all the microorganisms listed as good degrader of ammonium, *Nitrosomonas europaea*, the model chemolithoautotrophic ammonium oxidizing bacterium (AOB), obtains energy from the aerobic oxidation of ammonia (NH_3_) to nitrite (NO_2_^−^) [4]. Carbon dioxide (CO_2_) was used as the preferred assimilative carbon source via the Calvin-Benson-Bassham cycle for *N.europaea*’s growth and maintenance [5]. A significant amount of the energy obtained from oxidation of hydroxylamine has to be invested in ammonia oxidation to hydroxylamine, reverse electron transport to generate Nicotinamide adenine dinucleotide (NADH) and CO_2_ fixation. As a consequence, the growth rate and yield of *N.europaea* are relatively low [6].

To successfully apply this bacterium to the biological removal of ammonium, large quantities of biomass need to be produced. The growth of *N.europaea* and its ammonium oxidation process are believed to be extremely affected by variously nutritional and environmental conditions [7], [8]. For instance, the physiological growth state of *N.europaea* and the efficient substrate utilization were influenced by NH_4_^+^, dissolved oxygen and NO_2_^−^ according to Yu and Chandran [9]. Meanwhile, the production of nitrous from aerobic oxidation of ammonia constantly decreases the pH, and therefore the regular pH steps are required to neutralize the production of nitrous in the culture [10]. Previous improvements have been done by manipulating the nutritional parameters and physical parameters, such as, temperature [11], amino acids [12], and oxygen concentration [13]. The range of carbon sources capable of supporting growth has also been extended to include pyruvate [14] and fructose [15]. Moreover, metallic ions can lead to the reprogramming of cellular metabolic network during fermentation. The addition of Mg ^2+^ [16], Cu^2+^ and Fe^3+^ [5] is found to increase the activity of the ammonia oxidation.

The optimization of culture condition by single dimensional search is laborious and time consuming, especially for a large number of variables and it does not ensure desirable conditions [17]. Statistical experimental design techniques are useful tools for developing, improving, evaluating and optimizing biochemical and biotechnological process based on only a small number of experiments. In this paper, a novel integrated statistical design, which incorporated Plackett-Burman design, path of steepest ascent, response surface methodology (RSM) and multi-objective optimization will hopefully provide a valuable approach for optimizing the ammonium biological removal technology in waste water treatment.

## Materials and Methods

### 1. Microorganism and Preparation

*N.europaea* (ATCC 19718) suspensions were prepared by transferring a fresh −80°C frozen cell stock to minimal growth medium (ATCC medium 2265) containing 45 mM NH_4_Cl. The pH was adjusted to 8.0±0.1 via periodic addition of sterile 10 N NaOH. The cultures were grown in the dark at 26°C shaking at 100 rpm for 72 h. Cells were harvested by centrifugation (9 000 rpm, 30 min, 4°C), and washed twice with mineral medium without ammonium. The cell pellet was re-suspended in 40 mM KH_2_PO_4_ buffer (pH 7.8) at a concentration of 5×10^9^ cells/ml with an average viability of 98%. Purity of the culture was checked by periodically plating onto Luria broth agar plates.

### 2. Growth of Bacterium

Batch experiments were conducted in 250 ml Erlenmeyer flasks containing 100 ml of liquid medium. The composition of medium used in PBD experiments was described as Table 1. Trace element solution (TES) included (g/L): 10 mg of Na_2_MoO_4_·2H_2_O, 172 mg of MnCl_2_·4H_2_O, 10 mg of ZnSO_4_·7H_2_O, 0.4 mg of CoCl_2_·6H_2_O [9]. Initial pH was adjusted to 7.8 using 10 N NaOH. Phenol red (0.0003% final concentration) was used as pH indicator as described elsewhere [18]. All the flasks were heat-sterilized by autoclaving at 121°C at 103 kPa for 15 min prior to inoculating in a shaking incubator, in the dark. Cell concentrations at the beginning of each experiment was measured and normalized to ensure consistency among experiments. The pH of the culture was readjusted daily to pH 6.8–7.4 by the addition of the sterility 1 M NH_4_HCO_3_ or 1 M NaHCO_3_. All batch reactor conditions were run in triplicate. Each data point was expressed by an average with an error bar (i.e. standard deviation from three independent samples).

**Table 1:** Plackett-Burman experimental design for screening of the culture conditions.

Run	Factors^a^																		Responses^b^			
	A	B	C	D	E	F	G	H	I	J	K	L	M	N	O	P	Q	R	S	*C_B_*	*Y_N_*	*R_A_*
1	50	50	25	5	0	0.50	0.12	2	8	0.004	0.006	0.001	0.003	1	5	1	150	26	48	0.711±0.047	0.42±0.04	91.96±0.28
2	80	50	25	0	0	0.50	0.12	4	4	0.002	0.003	0.003	0.003	1	5	1	100	30	96	1.214±0.068	1.38±0.21	96.62±0.69
3	50	25	50	0	0	0.50	0.12	4	8	0.004	0.006	0.001	0.001	2	5	−1	100	30	96	1.195±0.101	1.02±0.08	93.15±0.26
4	80	25	50	5	0	0.25	0.12	4	8	0.004	0.003	0.003	0.003	1	10	−1	100	26	48	1.038±0.057	1.62±0.24	94.94±0.52
5	80	25	25	5	5	0.25	0.06	4	8	0.002	0.006	0.001	0.003	2	5	1	100	30	48	1.051±0.063	1.43±0.16	92.09±0.32
6	80	25	50	0	0	0.50	0.06	2	8	0.002	0.006	0.003	0.001	1	10	1	150	30	48	1.132±0.077	0.82±0.06	92.87±0.56
7	80	50	50	5	5	0.50	0.12	2	4	0.002	0.006	0.003	0.001	2	5	−1	100	26	48	0.971±0.037	1.25±0.17	92.89±0.74
8	50	25	25	5	0	0.25	0.12	4	4	0.002	0.006	0.003	0.001	2	10	1	150	26	96	0.633±0.041	0.22±0.03	89.84±0.18
9	50	50	25	0	5	0.50	0.06	4	8	0.004	0.003	0.003	0.001	2	10	1	100	26	48	0.816±0.044	0.48±0.06	90.46±0.37
10	80	25	25	0	5	0.50	0.12	2	8	0.002	0.003	0.001	0.003	2	10	−1	150	26	96	0.774±0.042	0.29±0.07	92.37±0.21
11	80	50	25	0	5	0.25	0.12	4	4	0.004	0.006	0.001	0.001	1	10	−1	150	30	48	0.735±0.042	1.53±0.20	93.96±0.43
12	50	50	50	0	5	0.25	0.06	4	8	0.002	0.006	0.003	0.003	1	5	−1	150	26	96	0.512±0.051	0.35±0.04	94.07±0.26
13	80	50	25	5	0	0.25	0.06	2	8	0.004	0.003	0.003	0.001	2	5	−1	150	30	96	0.810±0.047	0.26±0.02	91.51±0.63
14	80	50	50	0	0	0.25	0.06	2	4	0.004	0.006	0.001	0.003	2	10	1	100	26	96	1.172±0.157	1.13±0.16	92.37±0.14
15	50	25	25	5	5	0.50	0.06	2	4	0.004	0.006	0.003	0.003	1	10	−1	100	30	96	1.397±0.073	0.62±0.07	94.67±0.48
16	50	25	50	0	5	0.25	0.12	2	4	0.004	0.003	0.003	0.003	2	5	1	150	30	48	0.632±0.060	0.56±0.06	89.68±0.33
17	50	50	50	5	0	0.50	0.06	4	4	0.002	0.003	0.001	0.003	2	10	−1	150	30	48	0.493±0.049	0.38±0.04	88.11±0.24
18	50	50	50	5	5	0.25	0.12	2	8	0.002	0.003	0.001	0.001	1	10	1	100	30	96	1.412±0.128	0.98±0.11	95.28±0.53
19	80	25	50	5	5	0.50	0.06	4	4	0.004	0.003	0.001	0.001	1	5	1	150	26	96	1.036±0.054	0.88±0.07	95.82±0.86
20	50	25	25	0	0	0.25	0.06	2	4	0.002	0.003	0.001	0.001	1	5	−1	100	26	48	0.732±0.047	0.74±0.08	91.23±0.34

a A, NH_4_Cl (mM); B, Fructose (mM); C, Pyruvate (mM); D, L-Aspartic acid (mM); E, Arginine (mM); F, MgSO_4_ (mM); G CaCl_2_ (mM); H, K_2_HPO_4_ (mM); I, HEPES (mM); J, EDTA-Fe^3+^ (mM); K, FeSO_4_ (mM); L, CuSO_4_ (mM); M, ZnSO_4_(mM); N, TES (ml); O, Inoculation size (×10^6^ cells/ml); P, pH control (NaHCO_3_ (−1), NH_4_HCO_3_ (+1)); Q, Agitation speed (rpm); R, Temperature (°C); S, Time (h).

b *C_B_*, Biomass concentration (×10^8^ cells/ml); *Y_N_*, Nitrite yield (mg/mg); *R_A_*, Ammonium removal (%).

All experiments were performed in triplicate. Each response was represented as mean ± SD (n = 3).

### 3. Analytical Methods

Cells counts were performed by light microscopy using a Helber chamber (standard deviation [SD], 5%) [19]. Biomass concentration (*C_B_*, cells/ml) was calculated as the number of cells per volume of fermentation mash. Ammonium and nitrite were determined spectrophotometrically according to the stander method [20]. The cellular protein content was quantified by the Micro BCA Protein Assay Kit (Rockford, Illinois) according to manufacturer’s instructions after cells were collected by centrifugation at 1 000 g ×10 min, rinsed with MilliQ water to remove salt, and digested in 3 N NaOH at 65°C for 30 min. Nitrite yield (*Y_N_*, mg/mg) was calculated as a ratio of nitrite to the total cellular protein according to Shu et al [21]. Ammonium removal (*R_A_*, %) was determined by (ammonium _initial_ – ammonium _end_)/ammonium _total_.

### 4. Experimental Designs

#### 4.1. Screening of significant factors

Plackett-Burman design (PBD) is a highly effective technique which screens the critical factors that significantly influence the process and eliminates the insignificant factors from a large number of candidate factors [22]. This technique assumes that interactions among the factors will be much smaller than the important main effects. It is a fraction of a two-level factorial design (+1 or −1), which requires fewer runs than a comparable factional design and allows the investigation of n−1 factors in at least n experiments.

Here, 19 independent factors, with initial values determined by preliminary experiments based on the literature reviews, are shown in Table 1. The design matrix created by the statistical software package Minitab 16.0 (Minitab Inc., USA) is also represented in Table 1. The *C_B_*, *Y_N_* and *R_A_* were measured in triplicate and the averages were taken as the responses.

#### 4.2. Steepest ascent method

To approach the optimal range of the selected factors, steepest ascent method is used to move rapidly to the general vicinity of the optimum via experimentation [23]. This method constructs a path through the center of the design based on the coefficients from the PBD functions [24].

In this study, experiments for each response were performed along the path of steepest ascent with defined intervals, which were determined by the estimated coefficients and practical experience. The design and experimental results obtained are shown in Table 2. Once the path of ascent no longer led to an increase, the point would be near the optimal point and could be used as the center point for subsequent optimization.

**Table 2:** Experiment design of steepest ascent and corresponding response.

	Factors			Responses		
	NH_4_Cl concentration (mM)	TES (ml)	Agitation speed (rpm)	Biomass concentration(×10^8^ cells/ml)	Nitrite yield(mg/mg)	Ammonium removal (%)
Base point^a^	65	1.5	125			
Origin step unit^b^	15	0.5	25			
Slope^c^	+0.85	−1.45	−0.68			
Proportion^d^	+12.75	−0.725	−17			
New step unit^e^	+5.00	−0.28	−6.67			
New step unit witha demical	+5	−0.3	−7			
Experiment 1	65	1.5	125	0.772±0.084	0.62±0.02	90.45±1.04
Experiment 2	70	1.2	118	1.258±0.053	0.88±0.07	91.87±1.25
Experiment 3	75	1.0	111	1.383±0.075	1.34±0.15	93.11±0.98
Experiment 4	80	0.8	104	1.422±0.039	1.58±0.12	94.32±1.05
Experiment 5	85	0.6	97	1.326±0.112	1.21±0.20	92.01±0.77

a Zero level in the PBD in Table 1.

b Range of the unity level.

c Estimated coefficient ratio from Eq. (*Y_N_*).

d Origin step unit×slope.

e Proportion×0.392,where 0.392 is a factor determined by experimenter based on process knowledge or other practical consideration, and 0.392 is appropriate in his example.

#### 4.3. Optimization of significant variables using CCD

Response-surface methodology (RSM), which includes factorial design and regression analysis, helps in understanding the interactions among the factors at varying levels and selecting the optimum conditions for the design response [25]. This method has been widely used for the optimization of various processes in biotechnology.

In this study, the four selected independent factors were studied at five levels (−2, −1, 0, +1, +2) using the central composite design. A full 16 (2^4^) factorial design with 8 star points and six replication of the center points, resulting in a total number of 30 experiments, was performed based on the matrix built by the Design- Expert soft (version 8.0.4, Stat-Ease Inc., Minneapolis, USA). The coded and actual values of the factor as well as the design matrix for the 30 experiments are presented in Table 3.

**Table 3:** Central Composite Design (CCD) matrix, experimental data and predicted values by the response surface analysis.

Run	Factor^a^				Responses^b^					
	*C_N_* (mM)	*TES* (ml)	*AS* (rpm)	*T* (h)	*C_B_* (×10^8^ cells/ml)		*Y_N_* (mg/mg)		*R_A_* (%)	
	Coded (actual)	Coded (actual)	Coded (actual)	Coded (actual)	Actual	Predicted	Actual	Predicted	Actual	Predicted
1	0 (80)	0 (0.8)	0 (105)	0 (72)	3.038±0.000	3.198	1.79±0.00	1.91	98.13±0.00	97.56
2	1 (90)	1 (1.0)	1 (120)	−1 (60)	2.077±0.053	1.963	0.51±0.12	0.31	94.98±0.47	95.07
3	0 (80)	0 (0.8)	0 (105)	0 (72)	3.328±0.000	3.198	1.95±0.00	1.91	97.51±0.00	97.56
4	1 (90)	−1 (0.6)	1 (120)	−1 (60)	1.815±0.120	1.703	0.55±0.04	0.43	95.29±0.86	95.44
5	1 (90)	1 (1.0)	1 (120)	1 (84)	2.437±0.137	2.416	0.42±0.05	0.22	95.09±1.17	95.68
6	0 (80)	0 (0.8)	2 (135)	0 (72)	1.317±0.117	1.487	0.48±0.07	0.46	96.03±0.21	95.69
7	1 (90)	−1 (0.6)	−1 (90)	−1 (60)	2.385±0.032	2.228	1.26±0.10	0.98	97.68±0.98	97.77
8	−1 (70)	1 (1.0)	−1 (90)	−1 (60)	1.558±0.117	1.478	1.39±0.03	1.48	95.32±1.28	94.67
9	−1 (70)	1 (1.0)	1 (120)	1 (84)	1.233±0.085	1.226	0.47±0.09	0.57	95.61±0.73	95.93
10	1(90)	−1 (0.6)	−1 (90)	1 (84)	3.591±0.108	3.299	1.57±0.11	1.49	97.30±1.74	97.41
11	0(80)	0 (0.8)	0 (105)	2 (96)	2.857±0.084	3.155	1.05±0.05	1.25	97.15±1.45	96.60
12	−2 (60)	0 (0.8)	0 (105)	0 (72)	1.205±0.046	1.327	0.63±0.06	0.69	94.44±0.82	94.73
13	0 (80)	0 (0.8)	0 (105)	0 (72)	3.001±0.000	3.198	1.83±0.00	1.91	97.40±0.00	97.56
14	−1 (70)	1 (1.0)	−1 (90)	−1 (60)	1.808±0.028	1.814	0.47±0.04	0.61	95.45±0.37	95.50
15	1 (90)	1 (1.0)	−1 (90)	1 (84)	3.621±0.106	3.559	1.21±0.01	0.96	96.69±0.95	97.03
16	−1 (70)	−1 (0.6)	−1 (90)	−1 (60)	1.837±0.045	1.738	0.47±0.11	0.64	95.77±1.26	96.02
17	−1 (70)	−1 (0.6)	1 (120)	1 (84)	1.665±0.101	1.487	2.06±0.03	2.05	97.15±0.91	97.29
18	1 (90)	−1 (0.6)	1 (120)	1 (84)	2.418±0.120	2.156	1.78±0.09	1.71	96.21±0.75	96.06
19	0 (80)	0 (0.8)	0 (105)	0 (72)	3.071±0.000	3.198	1.97±0.00	1.91	97.34±0.00	97.56
20	−1 (70)	1 (1.0)	1 (120)	−1 (60)	1.526±0.082	1.507	0.35±0.03	0.66	93.97±1.30	94.12
21	0 (80)	0 (0.8)	−2 (75)	0 (72)	2.309±0.077	2.601	0.83±0.12	1.06	97.27±1.84	97.58
22	0 (80)	−2 (0.4)	0 (105)	0 (72)	1.062±0.052	1.254	1.58±0.08	1.78	97.17±0.71	97.30
23	1 (90)	1 (1.0)	−1 (90)	−1 (60)	2.608±0.107	2.489	2.04±0.14	1.82	97.91±1.09	97.39
24	0 (80)	0 (0.8)	0 (105)	0 (72)	3.343±0.000	3.198	1.93±0.00	1.91	97.25±0.00	97.56
25	2 (100)	0 (0.8)	0 (105)	0 (72)	2.668±0.083	3.007	0.54±0.08	0.69	96.55±0.82	96.22
26	−1 (70)	−1 (0.6)	−1 (90)	1 (84)	2.316±0.059	2.075	1.39±0.14	1.15	97.10±1.09	96.85
27	0 (80)	2 (1.2)	0 (105)	0 (72)	0.985±0.046	1.254	1.13±0.11	1.14	95.73±1.26	95.57
28	0 (80)	0 (0.8)	0 (105)	0 (72)	3.405±0.000	3.198	1.96±0.00	1.91	97.75±0.00	97.56
29	0 (80)	0 (0.8)	0 (105)	−2 (48)	2.203±0.112	2.366	0.82±0.04	0.83	94.64±0.69	95.15
30	−1 (70)	−1 (0.6)	1 (120)	−1 (60)	1.856±0.113	1.768	0.75±0.05	0.77	96.04±1.93	95.48

a *C_N_*, NH_4_Cl concentration; *TES*, the element solution; *AS*, Agitation speed; *T*, Fermentation time.

b *C_B_*, Biomass concentration; *Y_N_*, Nitrite yield; *R_A_*, Ammonium removal.

#### 4.4. Multi-objective optimization

The Multi-objective optimization (MOO) was applied when the second-order polynomial function for each objective was determined. Through a combination of entropy measurement technique (EMT) and weighted coefficient methodology (WCM), which is used to evaluate individual response weights on the basis of entropy of the entire process [26], which is employed to form a scalar objective function for finding solution for MOO [27], the overall *C_B_*, *Y_N_* and *R_A_* could be simultaneously optimized.

By using the EMT, the original sequence of all the three responses should be firstly normalized by the normalized value (r_pn_^*^) in higher-the-better type, according to Datta [26]. After entropy of each response (*h_n_*) was calculated by the distinguishing coefficient *ξ_ij_* and normalized coefficient *k*, the weight coefficient (*w_n_*) of each response was obtained.

The weight of each response should be real numbers like that *w_n_* >0 for all responses. The three second-order polynomial functions were normalized and aggregated. Pareto optimal set for the problem is generated by systematically varying the weighting parameters for the objective functions. A total maximum strategy was obtained by solving the aggregating of second-order polynomial function.

After the calculation of the weight coefficient of each objective, the relational coefficients (*Γ_n_*) were computed by the following equation:
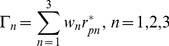
(1)


The higher the values of the relational coefficients are, the closer the corresponding factor combination is said to be the optimal.

### 5. Experimental Strategy

In this paper, culture condition of *N. europaea* leading to maximum biomass concentration (*C_B_*), nitrite yield (*Y_N_*) and ammonium removal (*R_A_*) was determined by a highly efficient integrated statistical design. The design comprised the following steps:(1) apply the Plackett-Burman Design (PBD) to identify the significant factors from 19 different factors; (2) use the path of steepest ascent for moving rapidly to the general vicinity of the optimum via experimentation; (3) employ the response surface methodology (RSM) in evaluating the significant factors and obtained optimization condition for each response; (4) employ the combination of weighted coefficient method with entropy measurement methodology in obtaining multi-objective optimization (MOO) condition for all the three desirable objectives; (5) carry out the confirmatory experiments under the optimized conditions to check the models.

### 6. Statistical Analysis

Variation due to model inadequacy was evaluated by Lack-of-fit test (LOF). The analysis of variance (ANOVA), which was carried out by Fisher’s statistical test (*F*-test), was employed for the determination of the significance of the models. Furthermore, the quality of the model was evaluated by the coefficient *R^2^*. The significance of the regression coefficient was tested via Student’s *t*-test.

## Results and Discussion

### 1. Screening of Significant Variables using Plackett-Burman Design

In the first step of this study, the influences of 19 independent factors on *C_B_*, *Y_N_* and *R_A_* were investigated using PBD. The experimental data of each response in Table 1 were correlated as first-order models and were shown in Table 4. The determinant of the coefficient *R^2^* of these models for *C_B_* (0.9352), *Y_N_* (0.9340) and *R_A_* (0.9559), indicated that the data variability could be explained by the models very well.

**Table 4:** Equations and the ANOVA results for the studied responses.

Response	Equations (with coded factors)	*R^2c^*	*Adj*.*R^2d^*	*Pred.R^2e^*
*PBD* a				
Biomass concentration	0.923+0.070A-0.024B-0.039C+0.036D+0.032E+0.010F+0.051G+0.008H-0.051I+0.022J+0.031K+0.028L-0.008M -0.069N +0.034O+0.058P-0.177Q+0.084R+0.092S	0.9352	0.8737	0.7410
Nitrite yield	0.81+0.24A-0.01B+0.01C+0.08D-0.02E+0.02F-0.06G+0.11H+0.11I-0.05J+0.03K+0.06L-0.06M-0.12N-0.02O+0.02P-0.49Q+0.16R-0.21S	0.9340	0.8713	0.7360
Ammonium removal	92.70+0.85A-0.01B+0.03C+0.22D+0.02E+0.43F+0.20G+0.38H+0.21I+0.18J+0.16K+0.09L+0.06M-1.45N-0.21O +0.01P -0.68Q+0.10R+0.88S	0.9559	0.9139	0.8234
*RSM* b				
Biomass concentration	3.20+0.42*C_N_* -0.28*AS* +0.20*T* +0.13*C_N_*×*TES* -0.14*C_N_*×*AS* +0.18*C_N_*×*T* -0.15*AS*×*T* -0.26*C_N_* ^ 2^-0.49*TES* ^2^-0.29*AS* ^2^-0.11*T* ^2^	0.9464	0.9136	0.7996
Nitrite yield	1.91-0.16*TES* -0.15*AS* +0.10*T* -0.17*C_N_*×*AS* -0.24*TES*×*AS* -0.34*TES*×*T* +0.19*AS*×*T* -0.30*C_N_* ^ 2^ -0.11*TES* ^2^ -0.29*AS* ^2^ -0.22*T* ^ 2^	0.9342	0.8939	0.7745
Ammonium removal	97.56+0.37*C_N_* -0.43*TES* -0.47*AS* +0.36*T* +0.24*C_N_*×*TES* -0.44*C_N_*×*AS* -0.30*C_N_*×*T* +0.25*AS*×*T* -0.52*C_N_* ^ 2^-0.28*TES* ^2^ -0.23*AS* ^2^ -0.42*T* ^ 2^	0.9115	0.8491	0.6632

a A, NH_4_Cl (mM); B, Fructose (mM); C, Pyruvate (mM); D, L-Aspartic acid (mM); E, Arginine (mM); F, MgSO_4_ (mM); G CaCl_2_ (mM); H, K_2_HPO_4_ (mM); I, HEPES (mM); J, EDTA-Fe^3+^ (mM); K, FeSO_4_ (mM); L, CuSO_4_ (mM); M, ZnSO_4_ (mM); N, TES (ml); O, Inoculation size (×10^6^ cells/ml); P, pH control (NaHCO_3_ (−1), NH_4_HCO_3_ (+1)); Q, Agitation speed (rpm); R, Temperature (°C); S, Time (h).

b *C_N_*, NH_4_Cl concentration (mM);*TES*, trace element solution (ml);*AS*, Agitation speed (rpm); *T*, Time (h).

c *R^2^*, coefficient of determination.

d *Adj*.*R^2^*, Adjusted *R^2^*.

e *Pred.R^2^*, Predicted *R^2^*.

Probability (*P*) values were used to check the significance of the coefficients, which are necessary to understand the pattern of the mutual interactions of the test factors. A smaller magnitude of the probability means a more significant correlation coefficient. The significance of the regression coefficient was tested with the confidence of 95%, so *p*≤0.0001 meant very significant; *p*≤0.05 was considered to denote a statistically significant difference and *p*≤0.01 also shown the power of significance.

**Figure 1:**
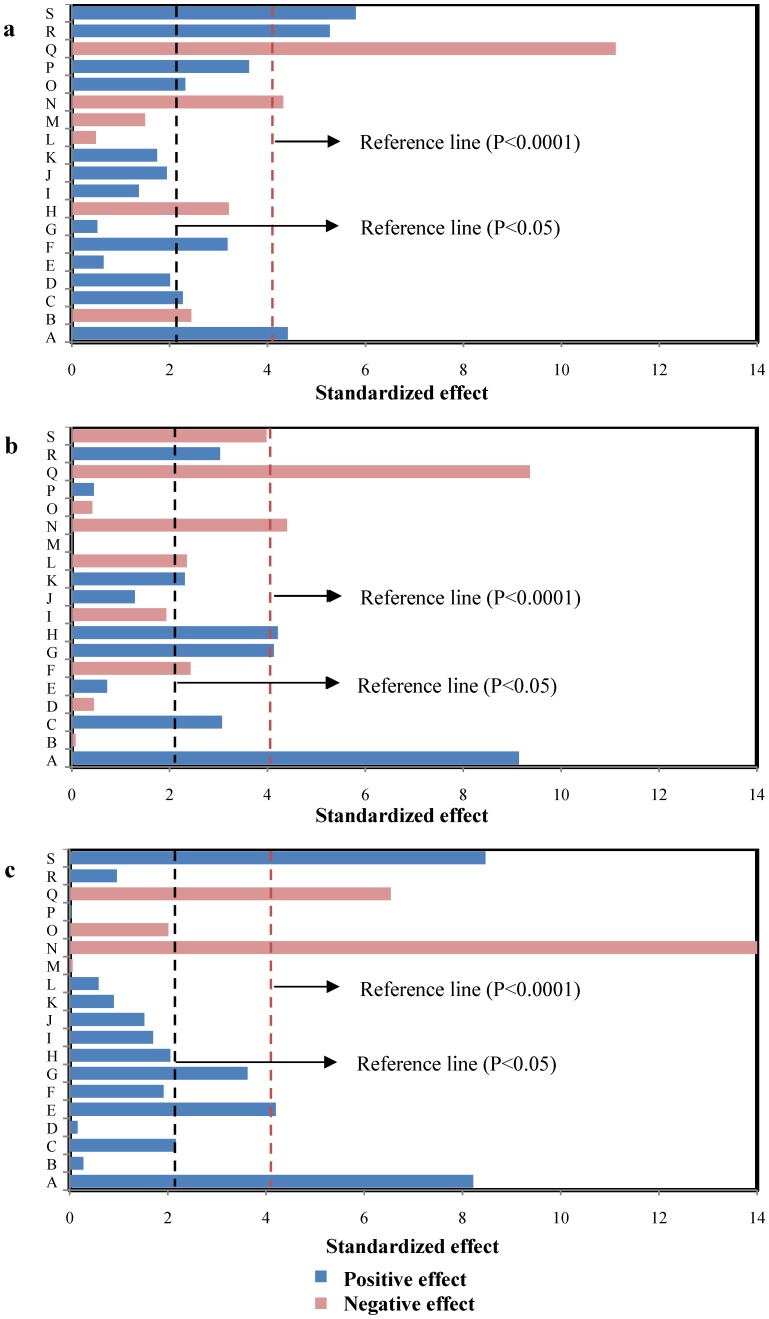
Standardized Pareto Chart showing the effects of the 19 independent factors on the three objectives.

Fig. 1 shows the effects of the variables on the response and significant levels. Based on the statistical analysis, the factors having the greatest impacts were identified and ranked as Q(*AS*)>S(*T*)>R(pH control) >A(*C_N_*)>N(*TES*) on *C_B_*, Q(*AS*) >A(*C_N_*)>N(*TES*)>H(CaCl_2_)>I(K_2_HPO_4_)>S(*T*) on *Y_N_* and N(*TES*)>S(*T*) >A(*C_N_*)>Q(*AS*)>F(Arginine) on *R_A_*. Thus, Q(*AS*), N(*TES*), A(*C_N_*) and S(*T*) were further optimized using RSM. Both Q(*AS*) and N(*TES*) had negative effects while A(*C_N_*) showed positive effects to all of the three responses. Meanwhile, the S(*T*) showed very significant positive effects on both *C_B_* and *R_A_*, but showed significant negative effects on *Y_N_*. This may have been due to the dissolved oxygen to the physiological growth of *N.europaea* [9].

### 2. Path of Steepest Ascent

Based on the aforementioned linear model equation (Table 1), the path of steepest ascent was determined to find the proper direction of changing the variables according to the sign of the main effects to improve *C_B_*, *Y_N_* and *R_A_*. Therefore, the path of the steepest ascent started from the center of the PBD and searched the proper direction to alter the levels of *TES*, *C_N_* and *AS* with other factors fixed at zero level.

The experimental design and corresponding results were shown in Table 2. The results indicated that the highest response was reached at the experiment 4 when *C_N_*, *TES* and *AS* were selected to be 80.0 mM, 0.80 ml and 104 rpm, respectively, which suggested that this level for each of the three factors was near the region of the maximum response.

### 3. CCD and RSM Analysis

Lack-of-fit tests (LOF) for deriving the best correlation between independent factors and responses were carried out. If a specific type of function, such as the linear function, the two-factor interaction(2FI) function, and the second-order polynomial function, adequately fits the data, as the largest portion of the error sum of squares is not due to lack of fit, the *p*-value should be large (>0.05). The “*p*-value” was 0.0027, 0.0001 and 0.0092 of the linear functions and was 0.0018, 0.0001 and 0.0112 of the 2FI functions for *C_B_*, *Y_N_* and *R_A_*, respectively. Both the linear functions and the 2FI functions had small *p*-values (<0.05) and should be cautiously used as the response predictor. By applying multiple regression analysis to the experimental data, the second-order polynomial function was established. Models that included insignificant factors were termed over-fitted, and were often unrealistically well fitted. Backward elimination is the used to improve the reliability of the RSM models. Therefore, the model terms, which *p*-values were lager than 0.05, have been omitted to give a better fit. As shown in Table 5, the “LOF *F*-value” of 1.92 (*C_B_*), 9.08 (*Y_N_*), and 2.21(*R_A_*), and the “LOF *p*-value” of 0.2429 (*C_B_*), 0.0119 (*Y_N_*), and 0.1960 (*R_A_*) were indicating that the “Lack of Fit” were not significant relative to the pure error.

**Table 5:** The analysis of the variance (ANOVA) for the quadratic models.

Source^a^	Biomass concentration (×10^8^ cells/ml)				Nitrite yield (mg/mg)				Ammonium removal (%)			
	df b	SS c	*F*-test	*p*-value	df b	SS c	*F*-test	*p*-value	df b	SS c	*F*-test	*p*-value
*C_N_*	1	4.23	78.71	<0.0001	–	–	–	–	1	3.35	16.90	0.0007
*TES*	–	–	–	–	1	0.62	15.87	0.0009	1	4.50	22.72	0.0002
*AS*	1	1.86	34.59	<0.0001	1	0.54	13.81	0.0016	1	5.38	27.16	<0.0001
*T*	1	0.93	17.37	0.0006	1	0.26	6.68	0.0187	1	3.13	15.82	0.0010
*C_N_*×*TES*	1	0.27	5.04	0.0375	–	–	–	–	1	0.96	4.83	0.0422
*C_N_*×*AS*	1	0.31	5.72	0.0279	1	0.47	11.85	0.0029	1	3.17	16.01	0.0009
*C_N_*×*T*	1	0.54	10.03	0.0053	–	–	–	–	1	1.42	7.18	0.0158
*TES*×*AS*	–	–	–	–	1	0.91	23.08	0.0001	–	–	–	–
*TES*×*T*	–	–	–	–	1	1.88	47.92	<0.0001	–	–	–	–
*AS*×*T*	1	0.38	7.08	0.0159	1	0.60	15.18	0.0011	1	0.96	4.85	0.0417
*C_N_* ^ 2^	1	1.82	33.84	<0.0001	1	2.52	64.16	<0.0001	1	7.44	37.59	<0.0001
*TES* ^2^	1	6.47	120.40	<0.0001	1	0.34	8.56	0.0090	1	2.16	10.93	0.0042
*AS* ^2^	1	2.28	42.44	<0.0001	1	2.24	56.97	<0.0001	1	1.46	7.38	0.0146
*T* ^2^	1	0.33	6.08	0.0239	1	1.28	32.47	<0.0001	1	4.86	24.54	0.0001
Model	11	17.08	28.87	<0.0001	11	10.04	23.21	<0.0001	12	34.68	14.60	<0.0001
Residual	18	0.97			18	0.039			17	3.37		
Lack of Fit	13	0.81	1.92	0.2429	13	0.052	9.08	0.0119	12	2.83	2.21	0.1960
Pure Error	5	0.16			5	0.006			5	0.53		
Cor Total	29	18.05			29	10.75			29	38.04		

a *C_N_*, NH_4_Cl concentration (mM); *TES*, the element solution (ml); *AS*, Agitation speed (rpm);*T*, Fermentation time (h).

b df, degree of freedom.

c SS, Sum of Squares.

Statistically significant at 95% of confidence level.

The results of ANOVA for the second-order polynomial functions are tabulated in Table 5. The “Model *F*-value” of 28.87 (*C_B_*), 23.21 (*Y_N_*) and 14.60 (*R_A_*) indicated that the models were significant. There was only a 0.01% chance that a “model *F*-value” this large could occur due to noise (*P*<0.0001) and most of the variation in the response could be explained by this regression equation. Meanwhile, the fit of the model was examined by the coefficient of determination *R* ^2^, which was 0.9464 for *C_B_*, 0.9342 for *Y_N_*, and 0.9115 for *R_A_*. It indicated that 94.64%, 93.42% and 91.15% of the variability in the responses could be explained by the models. The “predicted *R^2^*” of 0.7996, 0.7745, and 0.6632 were all in reasonable agreement with the “adjusted *R^2^*” of 0.9136, 0.8939, and 0.8491 for *C_B_*, *Y_N_* and *R_A,_* respectively. All the statistic results of the models show that the accuracy and general applicability of the second-order polynomial functions are adequate to describe the responses of experiments (Table 4).

By performing the significance test for each coefficient of the equation, it was found that the linear term of *C_N_* was shown very significant in *C_B_* (Table 5). Stein and Arp [28] reported that production of dense cultures of *N.europaea* with active cells must provide adequate ammonium. Meanwhile, the linear term of agitation speed (*AS*), which was interrelated to carbon source (CO_2_) supplied to the culture [10], also showed very significant effects on both *C_B_* and *R_A_* (Table 5)_._ The interaction terms of *TES*×*T* on *Y_N_* showed very significant effects. The quadratic terms, except for the *T* ^2^ on *C_B_*, *TES* ^2^ on *Y_N_* and *TES* ^2^, *AS* ^2^, *T* ^2^ on *R_A_*, also showed the very significant effects.

**Figure 2:**
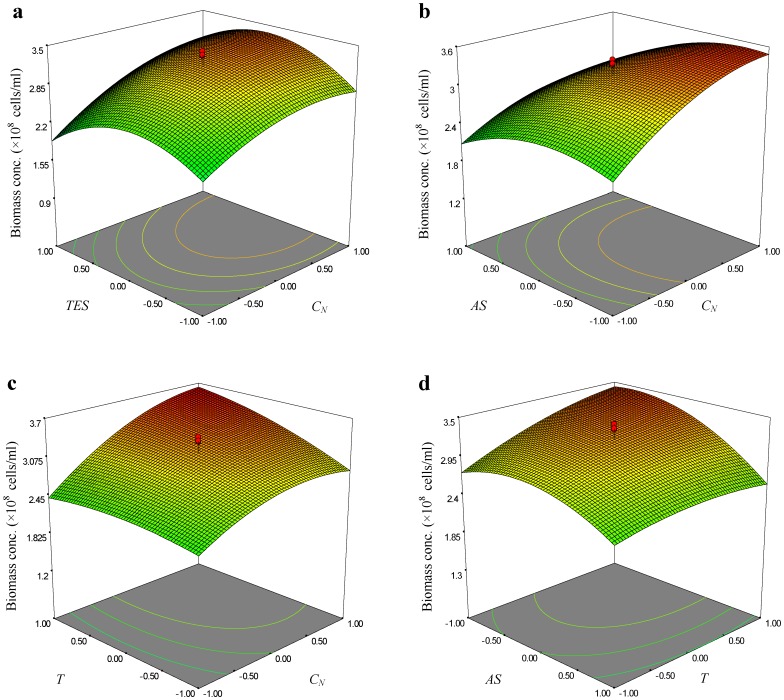
Response surface curve and contour plots of the quadratic model for biomass concentration.

**Figure 3:**
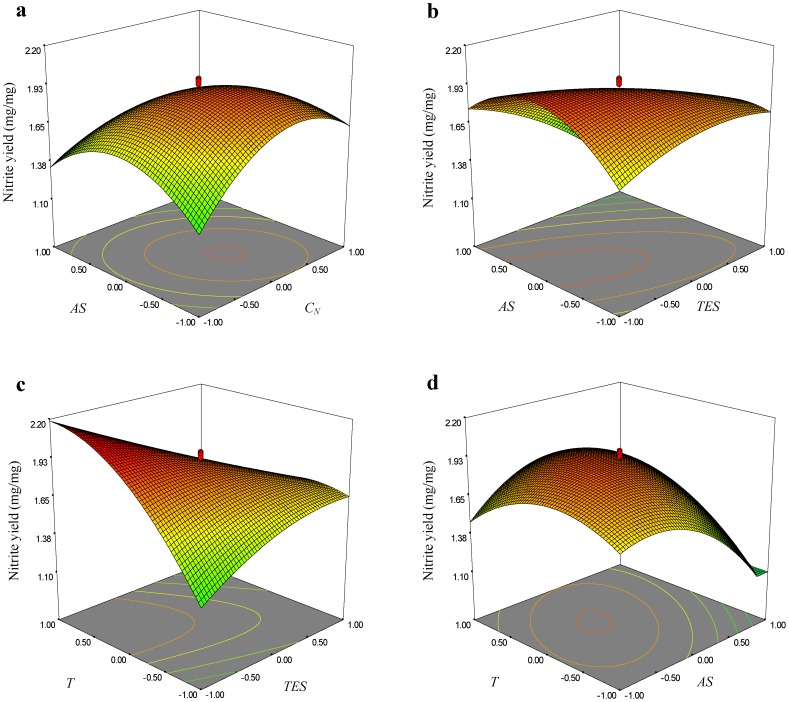
Response surface curve and contour plots of the quadratic model for nitrite yield.

**Figure 4:**
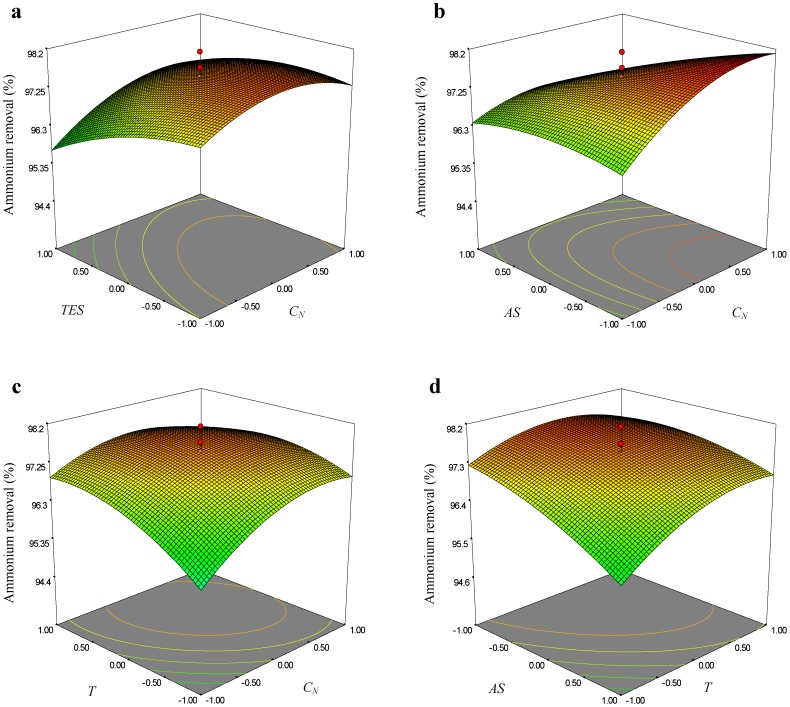
Response surface curve and contour plots of the quadratic model for ammonium removal.

The graphical representations of the regression model, were called the response surface plots and their corresponding contour plots were obtained using Design-Expert software and were presented in Fig. 2–4. The three-dimensional (3D) response surface curves were based on the second-order polynomial functions in which two variables were kept constant at their zero levels while the others varied. The shapes of the contour plot, such as elliptical or saddle, indicated that the mutual interactions between the variables were significant. The interaction terms of *C_N_*×*AS* and *T*×*AS* showed significant effects on all the three reposes. Fig. 2b, Fig. 3a and Fig. 4b showed the 3D response surface curves, the combined effect of *C_N_* and *AS* on the *C_B_*, *Y_N_* and *R_A,_* respectively. It revealed that at low and high levels of the *C_N_* and *AS*, the *Y_N_* was minimal. Meanwhile, responses of *C_B_* and *R_A_* were both increased with the increase of *C_N_* and the decrease of *AS*. The effects of *AS* and *T* on the responses of *C_B_*, *Y_N_* and *R_A_* at fixed *C_N_* and *TES* levels were shown in Fig. 2d, Fig. 3d and Fig. 4d, respectively. The curve and the curvature of the contour on the bottom indicated that at low and high level of the *AS* and *T,* the *Y_N_* was minimal. Meanwhile, it was observed that increasing the *T* drastically increased both the *C_B_* and *R_A_*, while increasing the AS drastically decreased both the responses.

The optimal conditions for maximum *C_B_*, *Y_N_* and *R_A_* were extracted by the Design Expert software through graphical model optimization and listed in Table 6. The maximum *C_B_*, *Y_N_* and *R_A_* were predicted to be 3.919×10^8^ cells/ml, 2.26 mg/mg and 97.86%, respectively.

**Table 6:** Response optimization using the response optimizer.

Objectives a	*w_n_* b	Code values				Conditions^c^				Predicted	Measured (mean ± SD)	*Γ_n_* d
		*C_N_*	*TES*	*AS*	*T*	*C_N_*	*TES*	*AS*	*T*			
*C_B_*	1.00	1.00	0.13	−0.99	1.00	90	0.83	90	84	3.919	3.886±0.072	0.463
*Y_N_*	0.00									1.30	1.33±0.06	
*R_A_*	0.00									97.45	97.46±0.96	
*C_B_*	0.00	−0.16	−1.00	0.52	1.00	78.4	0.60	113	84	2.428	2.491±0.025	0.338
*Y_N_*	1.00									2.26	2.18±0.08	
*R_A_*	0.00									97.52	97.06±1.09	
*C_B_*	0.00	0.72	−0.44	−1.00	−0.11	87.2	0.71	90	71	3.266	3.102±0.054	0.596
*Y_N_*	0.00									1.67	1.82±0.13	
*R_A_*	1.00									98.19	98.02±0.67	
*C_B_*	0.325	0.62	−0.15	−0.58	0.49	86.2	0.77	96	78	3.492	3.386±0.112	0.613
*Y_N_*	0.311									1.92	1.98±0.05	
*R_A_*	0.364									97.86	97.76±0.43	

a *C_B_*, biomass concentration (×10^8^ cells/ml); *Y_N_*, nitrite yield (mg/mg); *R_A_*, ammonium removal(%).

b *w_n_*, Weight coefficients on *n*th objectives.

c *C_N_*, NH_4_Cl concentration (mM); *TES*, trace element solution (ml); *AS,* agitation speed(rpm); *T*, time (h).

d *Γ _n_*, Relational coefficients on *n*th objectives.

### 4. Multi-objective Optimization

Through graphical model optimization, maximum *C_B_*, *Y_N_*, and *R_A_* were achieved under different optimal conditions. We need to find a multi-objective maximum strategy where the requirements simultaneously meet the critical properties. Thus, a compromise among the conditions for all the three responses is desirable. The multi-objective optimization method is used to achieve such a goal. The weighted coefficients method has been studied successfully for the optimization of multiple- response process [27].

Both *C_B_* and *R_A_* are significant indexes in the ammonium removal process, and both of them should be simultaneously taken into account when the overall ammonium removal process is evaluated. In addition, nitrite is the product of this process. Thus, *Y_N_* as an important index should also be considered. However, because of their different important degrees, weights of all the three response have to be determined. In this study, entropy measurement methodology was used to calculate the weight coefficients. After the experimental data in Table 3 were normalized, the calculated values of entropy for *C_B_*, *Y_N_*, and *R_A_* had been found as 0.912, 0.938 and 0.960, respectively. Therefore, the weights of *C_B_*, *Y_N_*, and *R_A_* are shown as: W = [0.463, 0.328, 0.209].

After three response functions were normalized and linear combining with the obtained weight coefficients, a MOO second-order polynomial response function was found. By solving this function, the maximum values of *C_B_* (3.492×10^8^ cells/ml), *Y_N_* (1.92 mg/mg), and *R_A_* (97.86%) were predicted to be obtained simultaneously. The optimized culture condition and its predicted response value were obtained and listed in Table 6. The relational coefficient (*Γ_n_*) for each strategy was also calculated using Eq.1. The *Γ_n_* of 0.613 indicated that MOO strategy had the best performance among the four strategies.

### 5. Model Validation

To confirm the adequacy of the model equations, confirmatory experiments under the optimized condition were carried out. All the confirmatory experiments were conducted in triplicate and the values predicted by the optimization model were set as controls.

In the single object maximum strategy, the *C_B_* of 3.886±0.072 ×10^8^ cells/ml reached 99.2% matching degree compared with the predicted values by the software (3.919 ×10^8^ cells/ml). The *Y_N_* obtained on the optimal culture condition was 2.18±0.08 mg/mg. It also reached 96.4% of the predicted value (2.26 mg/mg) under the same condition. The mean value of maximum *R_A_* was 98.02±0.67%, which also matched well with the predicted value (99.8%). All RSM models were successfully built with good validities.

In the MOO strategy, all the response values obtained by measuring in the optimal condition reach above 95% of the values predicted by the software under the same condition simultaneously. Therefore, the integrated statistical design strategies were successfully built with good validities. To the best of our knowledge, it is the first time of such high values of all the three objectives simultaneously obtained [1], [2].

### Conclusion

In this study, a four-step design, including the PBD, path of steepest ascent, RSM and WCM coupled with EMM for MOO, is used to optimize the culture condition of *N.europaea*. The novel method provides an attractive solution to simultaneously optimize four main influential variables (*C_N_*, *TES*, *AS* and *T*) on the *C_B_* and *Y_N_* as well as *R_A_*. Further confirmatory experiments demonstrate that such an integrated statistical design is an effective and powerful approach. In summary, the proposed integrated statistical design was useful for optimizing the ammonium removal process and held promise to be effective in wastewater treatment technology.
