# Correction: Application of an Integrated Statistical Design for Optimization of Culture Condition for Ammonium Removal by *Nitrosomonas europaea*

**DOI:** 10.1371/annotation/579cbc43-28e8-4e3d-a500-5b9d9793438b

**Published:** 2013-12-20

**Authors:** Bao yingling, Ye zhengfang

A grant from the National Natural Science Foundation of China was incorrectly omitted from the Funding Statement. The number of the grant is: NSFC 21261140336. 

